# Salicylic acid and jasmonic acid-mediated different fate of nickel phytoremediation in two populations of *Alyssum inflatum* Nyár.

**DOI:** 10.1038/s41598-024-64336-6

**Published:** 2024-06-10

**Authors:** Masoud Modarresi, Naser Karimi, Mehrdad Chaichi, Azam Chahardoli, Shiva Najafi-Kakavand

**Affiliations:** 1 Pharmaceutical Sciences Research Center, Health Institute, Kermanshah University of Medical Sciences, Kermanshah, Iran; 2 Laboratory of Plant Physiology, Department of Biology, School of Science, Razi University, Kermanshah, Iran; 3 Seed and Plant Improvement Research Department, Hamedan Agricultural and Natural Resources Research and Education Center, Hamedan, Iran

**Keywords:** *Alyssum inflatum*, Bioaccumulation factor, Jasmonic acid, Phytoremediation, Salicylic acid, Translocation factor, Plant sciences, Environmental sciences

## Abstract

This study investigates Ni phytoremediation and accumulation potential in the presence of salicylic acid (SA) (0, 50 and 200 μM) and jasmonic acid (JA) (0, 5 and 10 μM) in two populations of *Alyssum inflatum* under various nickel (Ni) doses (0, 100 and 400 μM). By measuring Ni levels in the shoots and roots, values of bioaccumulation coefficient (BAC), biological concentration factor (BCF) and translocation factor (TF) were calculated to quantify Ni accumulation and translocation between plant organs. Additionally, the amounts of histidine (His), citric acid (CA) and malic acid (MA) were explored. The results showed that plant dry weight (DW) [in shoot (29.8%, 8.74%) and in root (21.6%, 24.4%)] and chlorophyll [*a* (17.1%, 32.5%), *b* (10.1%, 30.9%)] declined in M and NM populations respectively, when exposed to Ni (400 μM). Conversely, the levels of MA [in shoot (37.0%, 32.0%) and in root (25.5%, 21.2%)], CA [in shoot (17.0%, 10.0%) and in root (47.9%, 37.2%)] and His [in shoot (by 1.59- and 1.34-fold) and in root (by 1.24- and 1.18-fold)] increased. Also, in the presence 400 μM Ni, the highest accumulation of Ni was observed in shoots of M (1392 μg/g DW) and NM (1382 μg/g DW). However, the application of SA and JA (especially in Ni 400 μM + SA 200 μM + JA 5 and 10 μM treatments) mitigated the harmful impact of Ni on physiological parameters. Also, a decreasing trend was observed in the contents of MA, CA, and His. The reduction of these compounds as important chelators of Ni caused a decrease in root-to-shoot Ni transfer and reducing accumulation in the shoots of both populations. The values of phytoremediation indices in both populations exposed to Ni (400 μM) were above one. In presence of the SA and JA, these indices showed a decreasing trend, although the values remained above one (BAC, BCF and TF > 1). Overall, the results indicated that SA and JA can reduce phytoremediation potential of the two populations through different mechanisms.

## Introduction

Heavy metals (HMs), like nickel (Ni), are among the most significant environmental contaminants. Large quantities of these pollutants have entered the environment due to increased industrialization and urbanization^1^. On the other hand, HMs naturally exist in the Earth's crust, but human activities have led to widespread environmental pollution^2^. In turn, HMs can enter the food chain via plants and pose severe risks to human health^3^.

In HM-rich soils such as ultramafic soils, plants use specific evolutionary mechanisms to tolerate and cope with HM toxicity. The most ordinary strategy is prevention, where the translocation of HMs taken up by the roots is limited to the aerial parts of plants, resulting in HMs accumulation in the roots^4^. In contrast, some plants can accumulate HMs within themselves, and the rapid evolution of this capability is well illustrated in HM-accumulators. These plants can gather HMs in their aerial parts, resulting in lower concentrations of HMs in their underground organs^5^. According to these strategies, plants retain genetic and physio-biochemical features to demonstrate themselves as the ultimate choice for remediation of HMs pollution^6^. Hyperaccumulator plants can collect HMs such as Ni, hundreds or thousands of times greater than typical for the most plants; However, the excessive presence of these HMs can cause toxicity to the majority of plants^7,8^. For example, to prevent Ni toxicity, hyperaccumulator plants such as *Alyssum murale*, store the largest amount of absorbed Ni in their leaf epidermal cells^7^. When normal plants are exposed to HMs such as Ni, they undergo numerous biochemical and physiological changes that negatively affect growth parameters and HM accumulation in their systems^9^. *Alyssum inflatum* Nyár. has been identified as a Ni-hyperaccumulating plant native to the Ni-rich serpentine regions of Western Iran.^10,11^. For the first time, Ghaderian et al. (2007) reported that *A. inflatum* can accumulate more than 3,700 (μg Ni g^−1^ shoot DW)^12^.

Ni as a transition metal, naturally occurs in soils in low amounts, except ultramafic soils, which are rich in Ni, cobalt (Co), and in some cases' manganese (Mn)^13^. Furthermore, low doses of Ni (approximately 10 mg Ni/kg plant tissue) are demanded for the development and metabolism of most plants. However, excessive quantities of Ni in plants lead to Ni toxicity, adversely impacting the physio-biochemical processes of the plants^14^. The physiological impacts of Ni toxicity on plants include a drop in biomass and alteration in photosynthetic capacity^15^. Most hyperaccumulator plants are Ni-accumulating species that typically grow in ultramafic soils^16^. In the last few decades, hyperaccumulating plants have been utilized to remove HMs, including Ni, from the environment through a process known as “phytoremediation”^17^. Fundamentally, phytoremediation focuses on two aspects: the ability of plants to accumulate HMs and the biomass of HMs in plants^18^. Among the important mechanisms for tolerating and preventing HMs toxicity in plants is the binding of HMs to high-affinity chelators, including amino acids, organic acids (OAs), nicotianamine (NA), glutathione (GSH), metallothioneins (MT), and phytochelatins (PCs)^6,19^. For the first time, Krämer et al. reported that high Ni concentration lead to increased histidine (His) contents in several Ni-hyperaccumulator species of *Alyssum*^20^. Additionally, the role of organic acids (OAs) is well-established in long-distance translocation of HMs from roots to shoots and in the accumulation of HMs in plant cells^19^. Mnasri et al. observed that Ni and cadmium (Cd) induced an increment in OAs levels, especially citric acid (CA) contents in the aerial parts and xylem sap of *Sesuvium portulacastrum*^21^. To assess a plant's potential for HMs phytoremediation, several parameters are use, including the biological concentration factor (BCF) and bioaccumulation coefficient (BAC), which evaluate the concentration of HMs in plant tissues relative to the soil, and translocation factor (TF), which assesses the capacity of HMs transport from roots to shoots^22^.

In recent decades, researchers have found that plant hormones such as Jasmonic acid (JA) and salicylic acid (SA) trigger signaling pathways that adjust growth and development, enhancing tolerance and mitigating the perilous effects posed by stress conditions such as HMs^23^. Therefore, the protecting action of SA against HM stress generally involves the adjustment of ROS levels and antioxidative mechanisms, induction of gene expression, and regulation of the uptake and distribution of elements^24^. Additionally, JA is considered a category of endogenous growth substance recognized in numerous plant species, which mediates multiple aspects of gene and metabolic fine-tuning, stress modulation, reproduction, defense, and cell signalling^25^. It has also been proven that the levels of endogenous SA and JA increase in reply to various stresses to mitigate the negative impacts of these stresses in plants^26^. Furthermore, in the presence of high Cd levels in the leaves of *Noccaea praecox*, as a Cd-hyperaccumulator plant, SA levels increased in aerial parts of this plant exposed to 50 mM Cd alone, while mechanical damage or fungal infection led to a rise in JA levels in the leaves of Cd-treated plants^27^. It appears that Cd increases the biosynthesis of indole-glucosinolate (GS), leading to the production of phenyl-GS and the biosynthesis of the antifungal compound sinalbin. Additionally, indole-GS also plays a role in auxin biosynthesis, resulting in an increase in its level. The reduction in the level of SA may be caused by the antagonistic interaction of auxin with the SA signaling pathway. Furthermore, boosting Cd concentration elevates the level of JA and stimulates the initiation of the JA/Ethylene signaling pathway in the defense response to fungal attack^27^. External treatment with SA in mustard (under Ni stress) and pepper (exposed to lead (Pb) stress) decreased root absorption and their accumulation in the leaves of these plants. It was found that SA prevented roots absorption of Ni and Pb and reduced their transfer from roots to shoots, and also led to the improvement of the antioxidant system and the glutathione-ascorbate cycle in these plants^28,29^. On the other hand, the external use of JA restricted the absorption of Ni by the roots and its transfer to leaves of soybean plants^30^. Similar results were observed in chromium (Cr)-treated *Brassica parachinensis* L. It was found that JA prevents the entry of Cr through the membrane into the cytoplasm by exerting a protective role on the root cell membrane. Additionally, JA stimulates the biosynthesis of pectin compounds, hemicellulose, and lignin, increasing the binding of Cr to the cell wall and limiting its absorption and entry into the root cells^31^. The current study focused on investigating the patterns of Ni accumulation and transport in metallicolous (M) and non-metallicolous (NM) populations of *A. inflatum* under the treatment of SA and JA. Therefore, SA and JA were used as regulatory factors to explore their role in reducing the effects of Ni toxicity and phytoremediation in the two populations of *A. inflatum*. The aim was to provide a clear understanding of the modulatory impacts of these phytohormones and their interaction on Ni stress in plants, as well as on the phytoremediation of contaminated soils by Ni-accumulating plants.

## Materials and methods

### Plant preparation and treatments

Seeds of *Alyssum inflatum* Nyár. of the M population were collected from serpentine sites in Marivan (35^°^ 13.625′ N; 46^°^ 27.184′ E), and the seeds of NM populations were gathered from non-serpentine of Shahu sites (34^°^ 56′ 47′′ N; 46^°^ 27′ 41′′ E), respectively from West of Iran in summer of 2015^10^ by Naser Karimi (Department of Biology, Razi University, Kermanshah, Iran) according to the institutional, national and international guidelines and laws. The permission to collect plants (license code: IR.KUMS.REC.1401.512) was obtained from the Pharmaceutical Sciences Research Center, Kermanshah University of Medical Sciences, Kermanshah, Iran, which identifies the institutional licensing committee that approves the collection of plants and experiments, including any relevant details. The plant populations were identified after being collected and transferred to the laboratory by Hosein Maroofi (Agricultural and Natural Resources Research and Education Center, Kurdistan, Iran). A voucher specimen of the plant was deposited in the dedicated herbarium of the Kurdistan Agricultural and Natural Resources Research and Education Center (No. 9562-HKS), which is available to the public upon request. All seeds were kept at 4 °C. To start the experiments, the surface of the seeds was first sterilized with 1% (*v/v*) sodium hypochlorite solution (NaOCl) for 10 min, then thoroughly washed with distilled water. They were planted in 162 pots with a volume of 450 mL [contained perlite: sand (2:1) mix], and each pot was placed in a bucket. A total of 81 pots were assigned to each population, and eight seeds from each population were planted in each pot. The seeds were watered with tap water for 15 days until germination, after which they were fed with a modified Hoagland solution according to the instructions of Najafi-Kakavand et al. (2019)^11^. Also, Hoagland’s solution for entire pots was replaced every 5-days. All pots were kept in the controlled conditions in a CG 72 Environ growth chamber [light (16 h, 25 °C)/dark (8 h, 16 °C) period, PPFD of 140 μmol m^−2^ s^−1^]. After 45 days of germination, all pots of each population were distributed in 27 treatment groups, and three pots were given to each treatment group. Applied treatments included different doses of Ni (0, 100 and 400 μM), JA (0, 5 and 10 μM), and SA (0, 50 and 200 μM) individually or in combination. Every 7 days, the treatments were applied alone or combined with Ni and SA (along with nutrient solution) and JA (by spraying the leaves) for 21 days. After the treatment period, all the *A. inflatum* plants in both populations were harvested and divided into the root and shoot segments for more investigation and held at − 70 °C.

### Dry weight measurement

At harvesting (21 days after exposure), root and shoot DW were measured. For this purpose, the separate parts of the shoot and root of each plant (three repetitions per treatment) were dehydrated in an oven at 75 °C for 72 h till stable weight.

### Chlorophyll content measurement

Extraction of photosynthetic pigments from leaf samples of two populations of *A. inflatum* was performed using Arnon (1949) method^32^. First, 100 mg of fresh leaf material was homogenized in 10 mL of cold 80% acetone, then centrifuged for 15 min (at 4000 rpm, 4 °C). The absorbance of chlorophyll *a* and *b* were assayed by spectrophotometer at A_663_ and A_645_ nm, respectively. Finally, the amount of photosynthetic pigment was estimated with the equations mentioned below:

$$\begin{gathered} {\text{Chl}}a\left( {{\text{mg}}/{\text{g leaves FW}}} \right) \, = \, \left[ {\left( {{12}.{7 } \times {\text{ A}}_{{{663}}} } \right) \, {-} \, \left( {{2}.{69 } \times {\text{ A}}_{{{645}}} } \right)} \right] \, \times {\text{ sample weight}} \hfill \\ {\text{Chl}}b\left( {{\text{mg}}/{\text{g leaves FW}}} \right) \, = \, \left[ {\left( {{22}.{9 } \times {\text{ A}}_{{{645}}} } \right) \, {-} \, \left( {{4}.{68 } \times {\text{ A}}_{{{663}}} } \right)} \right] \, \times {\text{ sample weight}} \hfill \\ \end{gathered}$$

### Ni content assayed

Ni levels in two parts (root and shoot) of both populations of *A. inflatum* were determined based on the method introduced by Ghasemi et al. (2009)^33^. Briefly, dried matter samples were digested with 60% nitric acid (2 mL) for 24 h at room temperature, followed by incubating at 90 °C. After 4 h of heating, the samples were cooled. Then, by adding 1 mL of H_2_O_2_, the test tubes containing the samples were again exposed to 90 °C in the water bath until the solutions were clarified. Finally, by adding deionized water, the final volume of each sample reached 10 mL. Ni analysis of shoots and roots was accomplished using atomic absorption spectrophotometry (AAS, Shimadzu model 6200).

### Phytoremediation potential

To assess Ni phytoremediation potential in two populations of *A. inflatum*, three parameters were used including:

*Biological concentration factor (BCF)* was estimated as the ratio of Ni contents (μg/g DW) in root tissue (*C*_*root*_) to Ni levels (μM) in roots’ environment solution (*C*_*solution*_)^34^, which was calculated as follows:

$${\text{BCF }} = C_{root} /C_{solution}$$

*Bioaccumulation coefficient (BAC)* was described as a ratio of Ni (μg/g DW) in the aerial part of a plant to the Ni contents (μM) in the roots’ environment solution^35^, which was calculated as follows:

$${\text{BAC }} = C_{shoot} /C_{solution}$$

A higher ratio points to better phytoaccumulation efficiency^36^.

*Translocation factor (TF)* was evaluated as the ratio between Ni concentrations (μg/g) in aerial parts (*C*_*shoot*_) to Ni concentration (μg/g) in roots (*C*_*root*_), which was calculated as follows:

$${\text{TF }} = C_{shoot} / \, C_{root}$$

TF > 1 indicates that *A. inflatum* plants transfer Ni effectively from the ground parts to aerial parts^37^.

### HPLC analysis

*Preparation of plant extract:* Using the ultra-sonication-assisted extraction (UAE) technique defined by Krishna et al. (2018), the plant extracts were prepared^38^. First, 5 mL of deionized water was added to 100 mg of dry-matter powder and homogenized by a sonication bath for 30 min. Homogenous samples were centrifuged (3000 rpm, 15 min) to obtain a clear plant extract. The supernatant was held in a cold place (refrigerator) and analyzed within 12 h of preparation.

*HPLC method*: The evaluation of citric acid (CA), malic acid (MA) and histidine (His) contents was performed by an isocratic mode at 1 mL/min flow rate with a Knauer HPLC instrument (equipped with Smartline 1000 quaternary pump version 7603, UV-detector 2600 version 7605, and Chrom Gate HPLC software 3.1.7) on an Eurospher 100-5 C18 column (250 × 4.6 mm, 5 μm) in the room temperature. The solvent system contained buffer phosphate (100 mM; pH 2.5). The samples' analysis was recorded at 210 nm. The volume of the injection for each sample was 40 μL. Three replicates were prepared from each sample. The chromatographic peaks of CA, MA and His in plant samples were certified by comparison of their retention time and UV-spectrum with those of the corresponding standards of referral to the standard. Measurements were made based on the linear calibration curves obtained from the standard solutions of CA and MA and His and the area under the curve of peaks of the standards and the plant extracts.

### Data analysis

A randomized complete block design (RCBD) with three replications was used to conduct a factorial experiment involving 4 factors to analyze variance. Factors in this experiment included different doses of Ni, SA and JA (mentioned in part 2.1) along with two distinct populations of *A. inflatum* seeds (M and NM). The LSMEANS statement (SAS ver. 9.4) was applied when the interaction between treatments was significant. All the values are presented in the tables and figures as mean ± SE (standard error). Principal component analysis (PCA) was performed using the fviz-pca function of the factoextra R package ver. 1.0.7^39^ to visually biplot treatments and variables.

## Results and discussion

### Shoot and root dry weight

As an essential element, plants need low quantities of Ni for optimal growth and development. However, higher concentrations of Ni cause toxicity and can lead to several harmful changes in plant physiology and anatomy^40^. In the present study, excess Ni doses can negatively affect plant growth; therefore, with increasing Ni concentration, a significant decrease was detected in the shoot and root DW of both populations of* A. inflatum*. The highest reduction in the shoot DW with 29.8% of M population and 21.6% and 24.4% in roots DW were observed in M and NM populations exposed to 400 μM Ni, respectively, compared to control (Table 1). One of the causes of reduced plant growth and biomass production in the presence of high concentrations of Ni is the lack of essential elements such as iron (Fe), copper (Cu), and manganese (Mn). Due to similar chemical properties, Ni competes with these elements for absorption through their transporters in the root^10^. Accordingly, Rathor et al. (2014) demonstrated a significant decline in dry matter yield in *Zea mays* plants due to the accumulation of elevated doses of Ni^41^. Another study showed that Ni at lower concentrations (0–5 mg Ni/L) induced a meaningful increment in dry matter yield, but at higher concentrations (6–25 mg/L) caused toxic effects and led to a significant decrease in dry matter yield^42^. However, the reduction in biomass of plants exposed to Ni stress is related to plant metabolism, photosynthesis, water relations, transpiration, disturbance in the absorption of the nutrient, and oxidative damage caused by Ni stress^43^. Based on Table 1, using SA and JA alone or in combination in Ni-stressed plants in both populations exhibited different effects on plant mass. Using of SA augmented shoot and root dry matter of M plants, as well as root DW of NM plants under Ni treatments. In contrast, JA decreased the shoot DW of both populations under Ni treatments. The SA plays a main role in the regulating of cell growth, seed germination, seedling development, and ion uptake and transport. JA influences root growth by preventing the primary root, forming of lateral roots, regenerating the root, and reducing adventitious root formation^23^. As shown in Table 1, the application of SA in high doses (200 μM) caused the highest DW in shoots (30.3%) and roots (27.5%) of M population plants under the highest Ni concentration relative to plants under 400 μM Ni stress alone. A similar result in the same treatment was displayed in the root region of the NM population (46.03%). Similarly, in strawberry plants, applying Ni (150–300 mg/L) with SA (2 mM*)* increased the DW and FW of shoots and roots^44^. Unlike SA, JA in the presence of high Ni concentrations did not induce significant alterations in shoot biomass of both populations. In contrast to this result, Azeem (2018) indicated that in *Z. mays* plants, JA at 6–10 μM increased the seedling emergence, leaf number, and shoot length alone and in combination with Ni (8 μM)^45^. According to this study, JA via elevating the action of antioxidant enzymes ameliorated the damaging impacts of oxidative stress on biomass production, growth, and protein amounts in Ni-stressed plants. However, JA treatment increased root biomass in plants against 400 μM Ni stress in both populations to plants against the same dose of Ni. The highest DW of the M population was detected in the presence of 400 μM Ni + 50 and 200 μM SA + 10 μM JA. In this condition, the shoots and roots DW were 1.16–1.23 and 1.13–1.31 times, respectively, more than the stressed plants under 400 μM Ni alone. However, an enhancement in biomass by simultaneous application of SA with JA was observed only in the roots of the NM population under high Ni toxicity. For example, the highest increase in root DW (40.4%) was detected in 400 μM Ni + 200 μM SA + 5 μM JA treatments concerning the stressed plants under 400 μM Ni alone. Based on Table 1, the maximum ratio R/S was detected in plants exposed to 400 μM Ni + 10 μM JA by 1.49- and 1.84-fold increment, in M and NM populations respectively, compared to untreated plants. In the same situation, the highest enhancement of this ratio was detected in the treatment of 400 μM Ni + 200 μM SA only in the NM population (1.84). Also, using SA and JA externally in plants against high Ni concentration only boosted the R/S ratio in the NM population by 1.54 compared to the control plant. Therefore, the positive impacts of SA and JA in improving the growth parameters of both populations of *A. inflatum* can be related to their role in the internal changes of phytohormone levels such as abscisic acid (ABA) in regulating the function of stomata and transpiration, enhancing the biosynthesis of photosynthetic pigments and ameliorating the antioxidant system^11^. Similarly, Zaid et al. stated that biomass of *Brassica juncea* L. decreased significantly in the presence of Ni stress particularly at 150 μM, relative to the control group. Still, co-application of Ni with SA augmented shoot and root biomass^28^. It has been reported that the exogenous application of JA at 0, 1, and 60 μM doses in sunflower seedlings growth media decreased the primary root development and reduced the number of lateral roots^46^. However, JA and SA act as essential signaling agents to adjust the defensive response in plants against abiotic stress, which can be due to the nature, exposure time, and intensity of stress in plants^23^.

**Table 1:** Changes in DW the presence of SA and JA in two parts of populations (NM and M) of *A. inflatum* plants stressed with various doses of Ni.

Ni	SA	JA	M plant species			NM plant species		
			Shoot dry weight (g)	Root dry weight (g)	Root/shoot ratio (%)	Shoot dry weight (g)	Root dry weight (g)	Root/shoot ratio (%)
0	0	Control	0.315 ± 0.010^cd^	0.074 ± 0.003^cd^	23.4 ± 0.61^g-n^	0.248 ± 0.006^g-m^	0.045 ± 0.004^s-v^	17.9 ± 1.35^s-x^
		5	0.217 ± 0.046^fi^	0.065 ± 0.003^g-k^	30.8 ± 6.90^a-d^	0.218 ± 0.007^g-o^	0.025 ± 0.003^z–c^	11.4 ± 1.29^y^
		10	0.270 ± 0.017^b^	0.063 ± 0.003^j-k^	23.4 ± 1.38^g-n^	0.248 ± 0.008^g-k^	0.033 ± 0.003^z-b^	13.3 ± 1.59^xy^
	50	0	0.356 ± 0.008^ab^	0.074 ± 0.004^cd^	20.8 ± 1.12^g-o^	0.247 ± 0.004^g-m^	0.036 ± 0.006^z-b^	14.2 ± 1.89^k-s^
		5	0.233 ± 0.071^b-f^	0.054 ± 0.002^o-p^	25.3 ± 10.2^f-i^	0.170 ± 0.005^h-t^	0.039 ± 0.002^v–y^	23.0 ± 1.56^hi^
		10	0.183 ± 0.041^b-k^	0.053 ± 0.003^o-q^	29.7 ± 6.52^b-f^	0.181 ± 0.009^h-r^	0.045 ± 0.004^s-v^	24.6 ± 3.18^g-l^
	200	0	0.325 ± 0.014^abc^	0.073 ± 0.003^de^	22.5 ± 1.73^g-n^	0.217 ± 0.008^l-o^	0.034 ± 0.006^z-a^	15.6 ± 2.95^u-y^
		5	0.259 ± 0.021^def^	0.062 ± 0.005^jk^	23.7 ± 0.18^a-d^	0.131 ± 0.008^l–t^	0.040 ± 0.004^v–y^	30.4 ± 1.79^bc^
		10	0.310 ± 0.033^cde^	0.074 ± 0.004^cd^	24.2 ± 3.90^g-m^	0.167 ± 0.010^l–t^	0.035 ± 0.003^z-b^	21.0 ± 2.32^j-r^
100	0	0	0.284 ± 0.010^def^	0.069 ± 0.004^efg^	24.2 ± 0.90^g-m^	0.261 ± 0.006^f-n^	0.048 ± 0.004^st^	18.1 ± 1.54^p-w^
		5	0.224 ± 0.039^def^	0.066 ± 0.002^f-i^	30.0 ± 3.97^bcd^	0.253 ± 0.010^f-n^	0.038 ± 0.002^x-b^	15.0 ± 1.04^w-y^
		10	0.214 ± 0.010^e-i^	0.068 ± 0.003^f-i^	31.7 ± 0.98^abc^	0.222 ± 0.008^k-p^	0.041 ± 0.002^u-x^	18.3 ± 1.30^p-w^
	50	0	0.290 ± 0.016^d-g^	0.076 ± 0.003^bcd^	26.3 ± 2.51^d-i^	0.231 ± 0.007^i-p^	0.038 ± 0.003^x-b^	16.2 ± 0.81^t-x^
		5	0.278 ± 0.045^d-g^	0.060 ± 0.003^klm^	22.1 ± 4.80^j-q^	0.213 ± 0.005^l-q^	0.052 ± 0.004^opq^	24.2 ± 1.19^g-l^
		10	0.280 ± 0.030^d-h^	0.065 ± 0.004^ghi^	23.3 ± 2.38^g-n^	0.179 ± 0.005^l-r^	0.034 ± 0.003^z-b^	18.6 ± 1.81^l–t^
	200	0	0.301 ± 0.017^d-h^	0.073 ± 0.004^de^	24.1 ± 1.00^g-m^	0.243 ± 0.011^i-m^	0.042 ± 0.005^u-x^	17.4 ± 2.641^s-x^
		5	0.296 ± 0.034^d-h^	0.066 ± 0.001^f-j^	22.5 ± 2.36^g-n^	0.190 ± 0.002^l-r^	0.051 ± 0.002^pq^	26.8 ± 0.51^d-h^
		10	0.339 ± 0.007^a-h^	0.078 ± 0.003^bc^	22.9 ± 0.99^h-p^	0.231 ± 0.006^j-p^	0.039 ± 0.003^w-a^	16.6 ± 1.37^vw^
400	0	0	0.221 ± 0.007^e–h^	0.058 ± 0.004^lmn^	26.3 ± 2.58^d-i^	0.226 ± 0.005^j-p^	0.034 ± 0.005^z-a^	15.1 ± 2.14^uv^
		5	0.190 ± 0.017^e-j^	0.062 ± 0.002^jk^	32.8 ± 2.63^ab^	0.231 ± 0.005^i-s^	0.051 ± 0.002^pqr^	22.1 ± 1.02^j-q^
		10	0.199 ± 0.033^e-j^	0.069 ± 0.002^e–h^	35.0 ± 5.75^a^	0.191 ± 0.007^n-s^	0.045 ± 0.002^stu^	23.6 ± 1.102^g-m^
	50	0	0.313 ± 0.009^c-h^	0.064 ± 0.004^jk^	20.3 ± 1.51^k-s^	0.212 ± 0.010^n-s^	0.054 ± 0.002^nop^	25.6 ± 0.75^e-i^
		5	0.308 ± 0.025^def^	0.056 ± 0.003^mno^	18.3 ± 2.29^p-w^	0.177 ± 0.005^o-s^	0.049 ± 0.003^qrs^	27.6 ± 2.05^c-g^
		10	0.386 ± 0.055^a^	0.072 ± 0.002^def^	18.9 ± 2.81^k-t^	0.174 ± 0.004^o-t^	0.035 ± 0.002^z-b^	19.8 ± 1.32^k-s^
	200	0	0.317 ± 0.011^c-h^	0.080 ± 0.002^ab^	25.3 ± 1.44^f-i^	0.192 ± 0.004^o-s^	0.063 ± 0.002^jk^	32.9 ± 0.95^ab^
		5	0.309 ± 0.048^d-h^	0.080 ± 0.005^ab^	26.2 ± 4.73^d-i^	0.213 ± 0.006^o-s^	0.057 ± 0.004^mno^	26.5 ± 2.45^d-h^
		10	0.363 ± 0.043^a^	0.084 ± 0.001^a^	23.3 ± 2.37^g-n^	0.194 ± 0.005^o-s^	0.043 ± 0.003^t-w^	22.1 ± 0.65^j-q^

All values in the table are presented as mean ± SE (n = 3).

### Photosynthetic pigments contents

**Figure 1:**
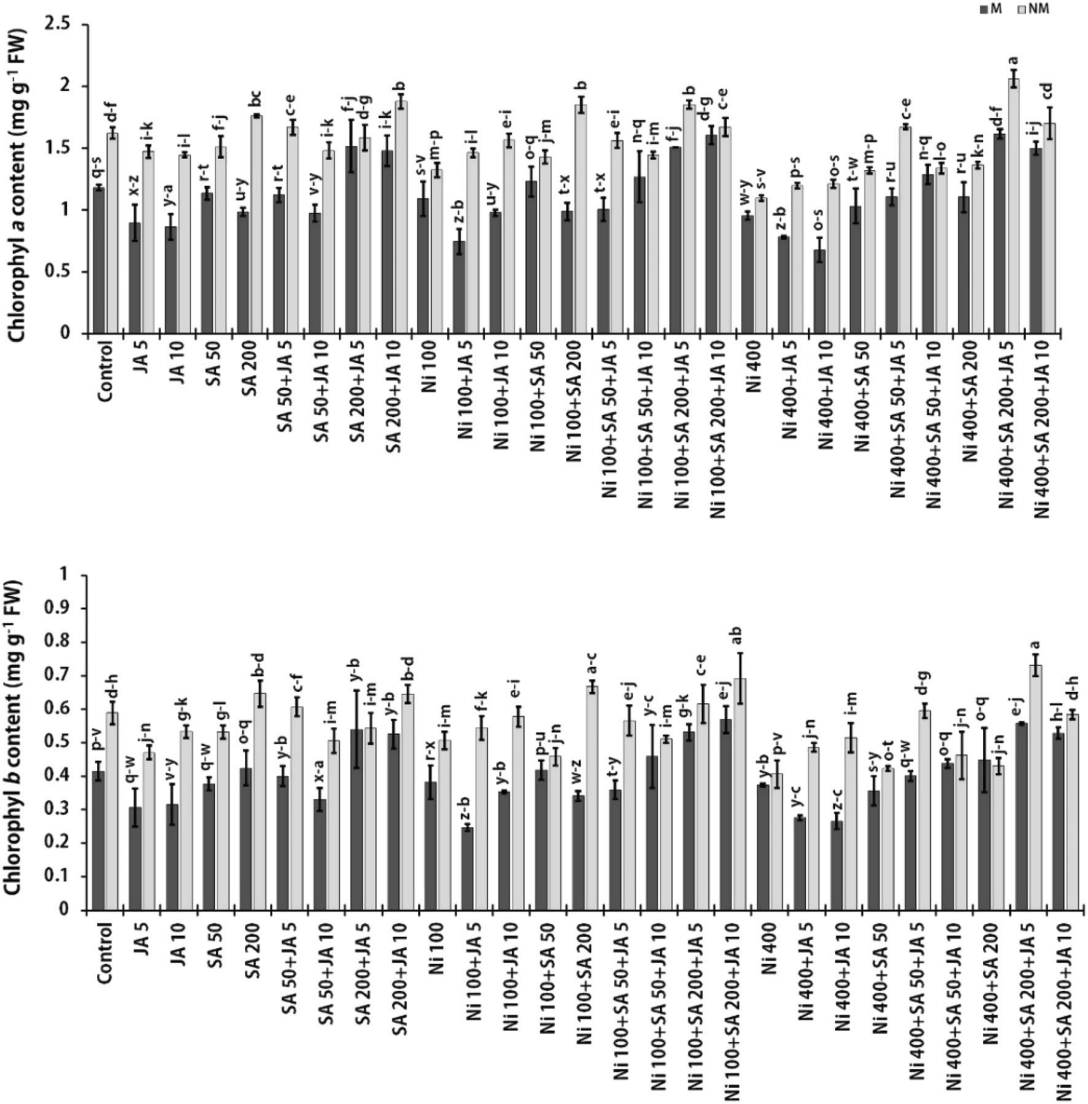
Changes in the amounts of chlorophyll *a* and *b* in the presence of SA and JA in two populations (NM and M) of *A. inflatum* stressed with various doses of Ni. All values in the figure are presented as mean ± SE (*n* = 3).

The destructive effect of Ni toxicity on physiological processes, including photosynthetic pigments, has been proven. As expected, augmenting Ni level led to a declining tendency in chlorophyll *a* and *b* content in both studied populations of *A. inflatum*. The Ni toxicity (at 400 μM) led to a decline in chlorophyll *a* concentration by 17.1% and 32.5%, and chlorophyll *b* content by 10.1% and 30.9% in the M and NM plants of *A. inflatum*, respectively, in comparison with the untreated plants (Fig. 1). Excessive amounts of Ni might directly destroy the photosynthetic machine of leaves via different routes. Excessive Ni can smash epidermal and mesophyll cells, disrupt thylakoid membranes and grana structures of chloroplasts, reduce grana size, and raise the number of lamellae in stressed areas. These alterations diminish chlorophylls, carotenoids, and xanthophylls amounts^14,47^. In fact, oxidative stress induced by elevating doses of Ni decreases the electron transport in the photosynthetic electron transport chain, a disorder in the activity of reaction centers (P680, and P700), and delays chlorophyll synthesis^48^. In line with this study, Ni at concentrations of 50 µM and 100 µM meaningfully declined the chlorophyll pigments (*a*, *b* and total) in cotton plants relative to the untreated plants^49^. Also, chlorophyll *a* and *b* contents in *Z. mays* plant leaves decreased after 13 days of plant growth under 100 and 200 mM Ni concentrations^50^. Similar to our results, Srivastava et al. showed that Ni considerably reduced the chlorophyll *a*/*b* ratio at concentrations of 0.1 and 1 mM, indicating that chlorophyll *a* was greater sensitive to Ni than chlorophyll *b*^51^.

The presence of SA or JA in both populations' growth media, especially at higher doses, led to a drop in the amount of chlorophyll *a* and *b* compared to control group. In this investigation, different impacts of SA and JA on chlorophyll content were evident in two *A. inflatum* populations against Ni toxicity. In both populations exposed to 400 μM Ni, SA, especially at high concentration (200 μM), caused a slight increase in chlorophyll content with respect to plants exposed to 400 μM Ni only (Fig. 1). One of the critical roles of SA is the regulation of photosynthetic pigments (chlorophyll and carotenoid), photosystem II (PSII) and the performance of carbonic anhydrase and Rubisco enzymes against HMs stress conditions^52^. It was also found that the co-treatment of SA and NO reduced Ni toxicity by improving photosynthetic apparatus, antioxidant enzymes' action, maintaining nutrients homeostasis and reducing MDA formation^53^. However, JA, especially at high concentration (10 μM) in M plants exposed to 400 μM Ni, showed a reducing trend of chlorophyll content. In contrast, the NM population treated with SA or JA alone with 400 μM Ni resulted in an augmenting trend of chlorophyll content compared to plants under Ni stress alone (Fig. 1). External application of JA improved the chlorophyll amount and development of *Glycine max* seedlings exposed to Ni stress^48^. Overall, the highest amounts of chlorophyll *a* and *b* in both populations were attended in the treatment of 400 µM Ni + 200 µM SA + 5 µM JA, which led to an enhancement in the amount of chlorophyll *a* (of 1.69- and 1.88 times) and chlorophyll *b* (of 1.49- and 1.06 times) in M and NM populations, respectively, compared to the plants treated with Ni (400 µM) alone (Fig. 1). Conferring to our results, the use of high doses of SA and JA leads to an increase in the chlorophyll *a* and *b* contents in both populations, which reduces the adverse impacts of Ni toxicity on the photosynthetic system by stimulating biosynthesis of photosynthetic pigments, reducing reactive oxygen species (ROS), and improving antioxidant system^11^. Accordingly, Kamran et al. reported that high doses of external JA alone inhibited the growth and photosynthesis rate in choy sum plants. Whereas, JA (5, 10, and 20 µM) mitigated chromium (Cr) toxicity (150 and 300 µM) and improved gas exchange and chlorophyll contents with respect to the Cr-alone treatment plants. In this study, the useful impact of JA was attributed to the protective roles of JA on the performance of the photosynthetic machine, biosynthesis of chlorophyll, guiding of stomata, and transport rates of CO_2_. These authors stated that reducing in Cr amounts in plant cells could be a significant reason for JA-induced stimuli on photosynthesis^31^. Furthermore, matching outcomes were detected in mustard plants in which Ni stress at concentrations of 50, 100, and 150 μM meaningfully decreased the amounts of chlorophyll by 22.15, 30.95, and 40.64%, respectively. In this study, the application of exogenous SA to Ni-stressed plants promoted chlorophyll amounts by 34.2%, 47.2% and 41.8% in the (50, 100 and 150 μM) Ni + SA treatments with respect to plants treated with Ni alone^28^, which confirmed the obtained results in the current work. In another study, the amount of photosynthetic pigments was significantly decreased in lead (Pb)-stressed maize plants as compared to non-exposed plants. Meanwhile, the exogenous usage of JA, SA, proline alone, and co-treatment with Pb significantly enhanced these pigment content in Pb-stressed and control maize plants. This was attributed to the protecting task of JA in enhancing the photosynthetic apparatus and uptake of key minerals against stress to Pb and the role of SA in falling chlorophyll disruption and MDA increase and electrolyte leakage in treated plants^54^.

### Plant Ni concentrations

To survey the impact of SA and JA on Ni uptake and potential phytoremediation, we determined the amount of Ni accumulated in the shoots and roots of Ni-exposed M and NM populations. As shown in Tables 2 and 3, with increasing doses of Ni in the plant root environment, the amount of Ni uptake by the plant roots in both populations showed a considerable increase. Thus, at 400 μM Ni treatment, the Ni doses in the ground part of the M and NM populations were 832 and 886 μg/g DW, respectively, which was 16.3- and 25.6 times higher than ground part of control plants. The uptake of Ni by ground part of plants occurred through two principal mechanisms, active transport system and passive diffusion pathway. Different factors such as plant species, Ni form, Ni amount in the rhizosphere, soil pH, organic matters in the soil, and plant metabolism affect Ni transport^40^. Furthermore, the uptake of Ni ions can be regulated by amino acids because these compounds act as chelators that form complexes with metal ions such as Ni, thus enhancing Ni uptake in plants^55^. The uptake of divalent cations in roots usually happens through apoplastic binding and symplastic uptake systems^56^. One study reported that the ratio of apoplastic to symplastic absorption in Ni-hyperaccumulator and non-hyperaccumulator plants was 85–95% to 5–15%, indicating that the symplastic route is very restricted for both plants^57^. Due to the rise in Ni uptake by the plant's roots, the rate of Ni transport from the root-to-shoot of these plants enhanced, therefore, excessive Ni accumulation was detected in the aerial parts of M and NM populations. After uptake, Ni is transferred simply into the xylem and then guided to aerial parts of plants^58^, then in there, Ni is redistributed through the phloem to stem, leaves, and other parts of plants^40^. The maximum Ni concentrations in the shoots of M and NM populations under Ni (400 μM) stresses were 1392 and 1382 μg/g DW, respectively, which was 88.2- and 71.6 times more than the aerial parts of untreated plants. The use of SA and JA alone and/or in combination in plants under Ni stress led to various accumulation patterns of Ni in the root and shoot of M and NM populations. Therefore, Ni uptake in root of the M population treated with the highest amounts of Ni, SA, and JA, i.e. 400 μM Ni + 200 μM SA and also 400 μM Ni + 10 μM JA was 12.6% and 38.4% respectively, with respect to plants treated with 400 μM Ni alone. However, in root of the NM population, the uptake of Ni at the highest dose of Ni and SA increased (6.24%), while it decreased (approximately 7.33%) against the highest dose of Ni and JA relative to the treated plants with 400 μM Ni alone. Furthermore, in M population plants treated with of 400 μM Ni + 200 μM SA + 10 μM JA, a higher Ni uptake (38.2%) was recorded in the root. While in the Ni-treated NM population root, the interaction of JA and SA led to the reduction in Ni uptake with the minimum amount at the treatments of 100 μM Ni + 50 μM SA + 5 μM JA and then at 400 μM Ni + 50 μM SA + 5 μM JA comparison with Ni treatments alone. Similar to our results, in soybean plants compared with leaves, a higher amount of Ni accumulated in plant roots against Ni stress with or without JA-priming. JA-priming in this plant eventuated in a lesser Ni accumulation in different parts of the plant, thereby lessening the damaging impacts of Ni and augmenting the growth yield of soybean. This was attributed to the stimulation role of JA in the production of OAs, for example, citrate and malate in root's exudates or thiol compounds to Ni sequestration^59^. On the other hand, Khalid et al. (2023) revealed that foliar 100 μM SA spray led to a decline in mercury (Hg) accumulation in the aerial parts, root, and fruit of *Capsicum annum* L. exposed to various doses of Hg (0, 50, 100 and 150 μM). Likewise, they confirmed that SA is important in controlling signaling molecules in developing and mediating plants' response to HM stress^60^.

**Table 2:** Changes in Ni levels and phytoremediation factors (BCF, BAC and TF) in the presence of SA and JA in the M population of *A. inflatum* stressed with various doses of Ni.

Plant species	Ni	SA	JA	Shoot Ni concentration (μg g^-1^ DW)	Root Ni concentration (μg g^−1^ DW)	BCF	BAC	TF
M plant species	0	0	Control	15.8 ± 2.89^y^	50.9 ± 4.03^s^	0 ± 0^q^	0 ± 0^t^	0.308 ± 0.03^v^
			5	6.90 ± 0.76^y^	45.2 ± 3.89^s^	0 ± 0^q^	0 ± 0^t^	0.153 ± 0.00^w^
			10	7.16 ± 0.49^y^	50.5 ± 4.34^s^	0 ± 0^q^	0 ± 0^t^	0.144 ± 0.02^w^
		50	0	27.3 ± 3.45^xy^	41.4 ± 5.04^s^	0 ± 0^q^	0 ± 0^t^	0.671 ± 0.14^l-o^
			5	21.3 ± 2.32^y^	41.4 ± 5.04^s^	0 ± 0^q^	0 ± 0^t^	0.517 ± 0.015^q-t^
			10	23.5 ± 1.55^xy^	54.4 ± 3.72^s^	0 ± 0^q^	0 ± 0^t^	0.432 ± 0.002^q–v^
		200	0	23.5 ± 1.55^xy^	54.4 ± 3.72^s^	0 ± 0^q^	0 ± 0^t^	0.432 ± 0.002^q–v^
			5	38.6 ± 0.87^xy^	60.1 ± 1.44^s^	0 ± 0^q^	0 ± 0^t^	0.643 ± 0.03^l-p^
			10	31.5 ± 1.10^xy^	55.5 ± 2.22^s^	0 ± 0^q^	0 ± 0^t^	0.57 ± 0.04^n-t^
	100	0	0	383 ± 8.87^m–o^	602 ± 18.0^l^	6.025 ± 0.185^d^	3.837 ± 0.089^ed^	0.638 ± 0.03^l-p^
			5	636 ± 34.5^j-k^	577 ± 13.9^ml^	5.774 ± 0.139^ed^	6.36 ± 0.345^b^	1.101 ± 0.034^w^
			10	565 ± 50.7^l^	538 ± 34.5^no^	5.38 ± 0.345^gf^	5.655 ± 0.508^b^	1.058 ± 0.16^d-g^
		50	0	317 ± 11.5^p-o^	553 ± 19.7^m–o^	5.528 ± 0.197^ef^	3.169 ± 0.116^g-i^	0.575 ± 0.042^n-t^
			5	148 ± 15.8^u-w^	798 ± 9.78^ij^	7.98 ± 0.098^c^	1.481 ± 0.158^r^	0.186 ± 0.018^w^
			10	329 ± 21.2^n-r^	839 ± 19.7^h^	8.386 ± 0.197^b^	3.29 ± 0.212^hg^	0.392 ± 0.017^v^
		200	0	248 ± 8.87^r-t^	563 ± 10.0mn	5.628 ± 0.1^ef^	2.477 ± 0.089^k-m^	0.44 ± 0.008^q–v^
			5	244 ± 7.88^r-t^	877 ± 28.0^g^	8.773 ± 0.289^a^	2.437 ± 0.079^mn^	0.279 ± 0.019^v-w^
			10	380 ± 9.37^m-p^	895 ± 6.00^g^	8.952 ± 0.617^a^	3.802 ± 0.094^d-f^	0.426 ± 0.029^q–v^
	400	0	0	1392 ± 173^a^	832 ± 29.1^hi^	2.08 ± 0.073^op^	3.481 ± 0.435^e–g^	1.671 ± 0.151^b^
			5	1269 ± 125^b^	1018 ± 13.0^e^	2.546 ± 0.034^j^	3.175 ± 0.313^g-i^	1.249 ± 0.14^cd^
			10	1163 ± 154^c^	1350 ± 45.8^a^	3.377 ± 0.115^ij^	2.907 ± 0.386^ij^	0.865 ± 0.144^h-j^
		50	0	1132 ± 52.7^c^	945 ± 15.1^f^	2.363 ± 0.038^mn^	2.831 ± 0.132^i-k^	1.198 ± 0.037^c-e^
			5	601 ± 195^kl^	1269 ± 16.2^c^	3.172 ± 0.041^ij^	1.503 ± 0.49^t^	0.476 ± 0.161^q-t^
			10	771 ± 3.45^e–g^	1310 ± 39.8^b^	3.276 ± 0.1^ij^	1.929 ± 0.009^n-q^	0.59 ± 0.021^l-r^
		200	0	853 ± 39.9^ef^	952 ± 38.0^f^	2.38 ± 0.098^mn^	2.134 ± 0.1^mn^	0.899 ± 0.074^g-j^
			5	669 ± 0.99^i-k^	1152 ± 34.3^d^	2.88 ± 0.086^k^	1.674 ± 0.003^p-r^	0.582 ± 0.019^n-s^
			10	822 ± 31.8^e–g^	1345 ± 16.9^ab^	3.364 ± 0.043^ij^	2.056 ± 0.08^on^	0.611 ± 0.016^l-q^

All values in the table are presented as mean ± SE (*n* = 3).

**Table 3:** Changes in Ni levels and phytoremediation factors (BCF, BAC and TF) in the presence of SA and JA in the NM population of *A. inflatum* stressed with various doses of Ni.

Plant species	Ni	SA	JA	Shoot Ni concentration (μg g^-1^ DW)	Root Ni concentration (μg g^-1^ DW)	BCF	BAC	TF
NM plant species	0	0	Control	19.3 ± 2.50^xy^	34.6 ± 3.74^s^	0 ± 0^q^	0 ± 0^t^	0.568 ± 0.134^n-t^
			5	56.5 ± 13.9^xy^	46.1 ± 4.07^s^	0 ± 0^q^	0 ± 0^t^	1.226 ± 0.283^c-e^
			10	64.8 ± 15.0^w-y^	50.8 ± 5.69^s^	0 ± 0^q^	0 ± 0^t^	1.31 ± 0.451^c^
		50	0	31.9 ± 5.50^xy^	30.8 ± 1.18^s^	0 ± 0^q^	0 ± 0^t^	1.036 ± 0.181^e–h^
			5	16.9 ± 0.50^xy^	30.8 ± 1.18^s^	0 ± 0^q^	0 ± 0^t^	0.549 ± 0.028^n-t^
			10	15.1 ± 4.90^y^	36.5 ± 4.036^s^	0 ± 0^q^	0 ± 0^t^	0.413 ± 0.126^q-w^
		200	0	15.1 ± 4.90^y^	36.5 ± 4.04^s^	0 ± 0^q^	0 ± 0^t^	0.413 ± 0.126^q-w^
			5	40.4 ± 0.60^xy^	53.5 ± 1.80^s^	0 ± 0^q^	0 ± 0^t^	0.756 ± 0.026^j-m^
			10	29.4 ± 0.35^xy^	35.2 ± 3.01^s^	0 ± 0^q^	0 ± 0^t^	0.841 ± 0.07^i-k^
	100	0	0	405 ± 11.1^mn^	520 ± 19.4^o^	5.202 ± 0.194^g^	4.049 ± 0.111^d^	0.78 ± 0.05^j-l^
			5	752 ± 101^g-i^	474 ± 10.0^p^	4.74 ± 0.10^h^	7.518 ± 1.01^a^	1.59 ± 0.247^b^
			10	661 ± 22.3^j-k^	336 ± 26.0^q^	3.36 ± 0.26^ij^	6.613 ± 0.223^b^	1.98 ± 0.221^a^
		50	0	296 ± 2.70^p-r^	353 ± 29.5^q^	3.535 ± 0.295^i^	2.961 ± 0.027^h-i^	0.842 ± 0.063^i-k^
			5	267 ± 38.0^r-s^	242 ± 16.0^r^	2.42 ± 0.160^mn^	2.674 ± 0.382^j-l^	1.116 ± 0.233^d-f^
			10	266 ± 23.2^q-r^	254 ± 25.5^r^	2.545 ± 0.255^lm^	2.658 ± 0.232^j-l^	1.046 ± 0.014^e–h^
		200	0	204 ± 17.8^s-u^	346 ± 38.0^q^	3.462 ± 0.386^i^	2.044 ± 0.178^no^	0.599 ± 0.115^l-r^
			5	164 ± 14.2^t-v^	259 ± 24.0^r^	2.59 ± 0.24^lm^	1.642 ± 0.142^q-r^	0.635 ± 0.004^l-p^
			10	105 ± 12.6^v-x^	273 ± 23.0^r^	2.73 ± 0.23^kl^	1.05 ± 0.126^s^	0.384 ± 0.014^t-v^
	400	0	0	1382 ± 21.8^a^	886 ± 19.5^g^	2.217 ± 0.049^np^	3.456 ± 0.055^fg^	1.56 ± 0.01^b^
			5	872 ± 30.6^e^	777 ± 62.0^j-k^	1.943 ± 0.155^p^	2.18 ± 0.077^mn^	1.13 ± 0.13^c-f^
			10	890 ± 16.0^e^	821 ± 8.50^hi^	2.054 ± 0.022^op^	2.226 ± 0.04^mn^	1.084 ± 0.031^d-g^
		50	0	823 ± 38.4^e–g^	952 ± 11.0^f^	2.38 ± 0.028^mn^	2.058 ± 0.097^no^	0.865 ± 0.031^h-j^
			5	982 ± 4.00^d^	743 ± 9.50^k^	1.859 ± 0.024^p^	2.455 ± 0.01^r^	1.321 ± 0.012^c^
			10	806 ± 111^f–h^	818 ± 37.5^hi^	2.047 ± 0.094^op^	2.016 ± 0.277^n-p^	0.991 ± 0.181^f-i^
		200	0	689 ± 15.2^h-i^	945 ± 21.5^f^	2.364 ± 0.054^mn^	1.724 ± 0.038^o-r^	0.73 ± 0.001^g-j^
			5	421 ± 7.30^m^	803 ± 2.00^h-j^	2.008 ± 0.063^op^	1.054 ± 0.019^s^	0.526 ± 0.026^q-t^
			10	346 ± 44.8^m-q^	900 ± 21.0^g^	2.25 ± 0.053^no^	0.864 ± 0.112^s^	0.385 ± 0.059^t-v^

All values in the table are presented as mean ± SE (*n* = 3).

As shown in Tables 2 and 3, external usage of SA in combination with JA or alone decreased Ni uptake in the shoot of Ni-stressed M and NM populations. In contrast, exogenous JA led to an increase in Ni absorption in the stressed plant shoot. The maximum reduction of Ni uptake in the aerial parts of M and NM populations was detected in the groups of 400 μM Ni + 200 μM SA (38.7% and 50.1%), and 400 μM Ni + 10 μM JA (16.5% and 35.6%), respectively, compared to plants treated with 400 μM Ni. Besides, a sharp reduction in Ni accumulation in the shoots of both populations was perceived with increasing doses of Ni under the effect of simultaneous treatment with SA and JA. Accordingly, the lowest shoot Ni accumulation was related to the 400 μM Ni + 50 μM SA + 5 μM JA in the stressed-M plants (with 56.8%) and 400 μM Ni + 200 μM SA + 10 μM JA in the stressed-NM plants (with 75%). According to our results, SA and JA, especially in high doses, led to a decrease in Ni absorption by the roots and a decrease in its accumulation in the shoots of both populations by a possible reduction in the expression of transporter genes involved in its uptake and translocation from root to shoot^11,61^. Accordingly, Ali et al. (2018) explained that amount of Cd in the aerial parts of *Brassica napus* increased in Cd stress conditions, but exogenous application of JA significantly decreased Cd accumulation in stressed plants. This study revealed that reduction in HMs absorption in reaction to external usage of stress phytohormones such JA, SA, abscisic acid (ABA), and brassinolide could be attributed to accumulation/ exudation of organic compounds^59^. Also, in another study, the mitigation of Cd ions uptake and protection of plants against HMs stress were attributed to the endogenous and exogenous SA in plants. SA pre-soaking inhibited the accumulation of Cd in the shoot as a result of a considerable reduction in roots BCF and Cd transport, which were calculated using the TF and BAC in shoots, respectively^62^. Nevertheless, the findings of the current study showed the role of SA and JA in Ni absorption and accumulation in stressed plants, which may indicate the formation of Ni- organic or amino acids complexes to the chelation and sequestration of Ni in the root and thus tolerance of plants against Ni toxicity.

### Phytoremediation potential

Phytoremediation is an internal mechanism for the absorption of HMs and deposition in their tissues, which allows the removal of HMs from contaminated sites at a higher rate and subsequent harvesting at a lower cost^63^. Generally, three main factors TF (shoot-to-root ratio of HMs), BCF (root-to-soil ratio of HMs), and BAC (shoot-to-soil ratio of HMs) are used to estimate the phytoremediation potential. Plants are expected to be appropriate for phytoremediation if the values of these factors are higher than one (TF > 1, BCF > 1, and BAC > 1)^8,35^. The averages of TF, BCF, and BAC values in different parts of M and NM populations of *A. inflatum* were shown in Tables 2 and 3, which can help to investigate the phytoremediation ability of two populations by explaining the characteristics of Ni accumulation and transport behaviors in them. The values of BCF and BAC of both populations under different Ni doses were higher than one (BAC > 1, BCF > 1), while the TF value was higher than 1 in high Ni concentration. The plants' roots had the highest BCF value at 100 μM Ni concentration [M population (6.025), NM population (5.202)]; however, a trend of decrease in BCF value was observed in M (2.08) and NM (2.217) populations of *A. inflatum* with the increase of Ni doses to 400 μM. Likewise, this decreasing trend was detected with the rise of Ni dose to 400 μM in BAC value with 9.3% and 14.6% in M and NM populations, respectively, in comparison to the groups treated with Ni (100 μM) alone. In addition, the TF value was less than 1 (TF < 1) in both populations at 100 µM Ni, reaching higher than 1 when the Ni dose increased to 400 µM [M population (1.671), NM population (1.56)]. Since the BCF, TF, and BAC values for Ni are greater than 1, two populations of *A. inflatum* could be used as Ni-hyperaccumulator plants for phytoremediation of Ni-contaminated soil. Similarly, it was found that the accumulation and translocation of Ni in various parts of *Alyssoides utriculata* found that BCF and TF are intensely more than 1 and this plant is a suitable candidate for Ni phytoremediation from serpentine soils^64^. Also, in an investigation performed by Sajad et al. (2020) to identify plants capable of phytoremediation of Ni from sixty-one sites in Pakistan, it was found that most plant species did not belong to the Ni hyperaccumulators group. Founded on the results of estimating the Ni doses in the soil, root and shoot, also analyzing the values of BCF, TF, and BAC, most of the species have the ability to Ni phytoremediation, for example, *Xanthium strumarium* (BCF: 89.97) suggested to phytostabilization and *Bryophyllum daigremontianum* (with TF: 2.37 and BAC: 198.11) suggested to phytoextraction^65^. Overall, the accumulation of HMs in plants relies on the outer environment and inner physiological characteristics, including HMs bioavailability, physicochemical characteristics of soil, biodiversity of microbes in soil, root exudation, temperature, and the existence of transporters^66^.

According to Tables 2 and 3, external application of SA and JA alone or in combination in plants against various concentrations of Ni, with rising Ni doses, a reduction trend was observed in the BCF and BAC indices of both populations of *A. inflatum*; however, BCF and BAC values of these treatments were greater than one. As an exception, exogenous JA use, especially at a lower concentration (5 μM) in plants under Ni (100 μM) stress, led to an enhancement in the BAC value of the M population (6.36) by 1.7 times and the NM population (7.518) by 1.9 times comparison with Ni (100 μM) treatment [M population (3.837), NM population (4.049)]. Also, the highest BFC value was recorded in the M population of *A. inflatum* in simultaneous treatments of 200 μM SA + 10 μM JA exposed to 100 μM (8.952) and 400 μM (3.364) of Ni in comparison with plants against Ni [100 (6.025) and 400 (2.08) μM] only. Unlike the roots, the BAC value displayed a decreasing trend in these treatments. Nevertheless, all these values are higher than one. Similarly, in the NM population treated with 400 μM Ni + 200 μM SA + 10 μM JA, the BFC value exhibited a slight increase, while in this treatment, the BAC value (0.864) decreased significantly with 74.9% compared to the plants under 400 μM Ni stress (3.456), that showed this value was less than one (BAC < 1). Besides, the TF values incremented in the presence of JA, especially at the higher dose (10 μM) in 100 μM Ni-treated plants in both populations (TF > 1), whereas at the same dose of Ni, the existence of SA led to a discount in the two populations (TF < 1). Generally, treatment with SA and JA in plants in the presence of 400 μM Ni resulted in a reduction in the potential of Ni transport to the aerial parts of the plant (TF value) in both populations. Therefore, the decreasing trend of TF values was shown in 400 μM Ni + 200 μM SA treatment [M population (0.899) and NM population (0.865)], and 400 μM Ni + 10 μM JA treatment [M population (0.73) and NM population (1.084)], in comparison to the plants under Ni (400 μM) stress [M population (1.671) and NM population (1.56)]. Similarly, simultaneous treatment of SA and JA in plants under 400 μM Ni resulted in the minimum Ni transport from the root-to-shoot of M and NM population plants (TF < 1). The minimum TF values were detected in 400 μM Ni + 200 μM SA + 10 μM JA treatment in the NM population (0.385), and 400 μM Ni + 50 μM SA + 5 μM JA treatment in the M population (0.476). Our finding indicates that SA and JA probably play an important role in altering the expression of genes involved in the absorption of Ni by the root, transfer to the shoots and its sequestration in the intracellular organelles of both *A. inflatum* populations by triggering signaling cascades. These phytohormones prevent Ni transpotr and accumulation in shoots by stimulating the decrease of expression of transporter genes involved in Ni absorption and also reducing the content of Ni chelators including His, organic acids and NA^11,25,61^. Similar to our results, Kazemi et al. (2010) informed that the exogenous use of SA in *B. napus* L. exposed to Ni toxicity caused a discount of Ni accumulation in the shoot. They suggested that SA probably prevents the accumulation of Ni in the aerial parts by reducing the root uptake and also reducing root-to-shoot Ni translocation^67^. Likewise, it was found that the callus cultures of two wheat genotypes, tetraploid (Durum-97) and hexaploid (Shafaq-06), which were under Cd toxicity, raised Cd accumulation in the callus with the augmentation of Cd doses in the medium. The usage of 0.5 mM SA led to decreased BCF value to less than 1 in higher concentrations of Cd^68^. Similarly, seed priming of *Linum usitatissimum* L. with SA reduced the amount of Cd in the root and shoot of this plant, and as a result, the values of BCF, BAC, and TF were reduced to less than one. In this way, SA prevented the toxicity of Cd in the plant by preventing the absorption of Cd and its transport to the shoot by xylem^62^. As confirmed by our results, Coelho et al. (2020) found that the use of JA resulted in the accumulation of arsenic (As) above 1000 μg/kg DW and enhanced BCF value (281) in *Lemna valdiviana*. They stated that JA leads to an enhancement in As accumulation in plants by modulating ROS homeostasis and signaling and promoting the antioxidant system^69^. On the contrary, the decreasing trend of Cd accumulation with significantly decreased BCF and TF values was observed in chickpea (*Cicer arietinum*) plants treated with JA and gibberellic acid (GA3) alone and/or in combination^70^. It has also been proven that exogenously JA treatment can disrupt the absorption, transport (root-to-shoot), and accumulation of HMs by suppressing the transporter genes involved in the absorption of HMs by root from soil such as As (*Lsi1*, *Lsi2,* and *Lsi6* genes) and Cd (*AtHMA4* and *AtHMA2* genes), as well as transporter genes related to HMs xylem loading such as Cd (*AtIRT1* gene), in plants^71^. As indicated by our results, in the existence of SA and JA, the BCF value had an increasing trend compared to the BCF of plants exposed to 400 µM Ni alone in plants under high Ni concentration. In contrast, the values of BAC and TF exhibited a decreasing trend. Accordingly, SA and JA can probably prevent the accumulation of Ni in the aerial parts of plant by stimulating the uptake, sequestration, and precipitation of Ni in special organelles of root cells, such as vacuoles^72^, as well as disrupting the mechanisms of Ni translocation from root to shoot.

### Citric acid (CA) and malic acid (MA) concentrations

Organic acids (OA) have multiple roles in plants; for example, MA, CA, and oxalate induce HMs tolerance by moving them via the xylem and sequestrating them in vacuoles^73^. According to Kocaman (2023), increasing the content of malonic acid and MA (up to 1 unit) in the plants can lead to binding (chelation) of 0.7 and 0.5 units of Ni^55^. In addition, OAs are chelated by their carboxyl groups with cationic HMs, thus reducing the amount of free toxic active ions. After entering, cationic HMs may stimulate OA channels or interact with a receptor protein and induce effective genes in the synthesis of OA. Occasionally, in the root surface, OA-HM complexes can arrive at cells through diffusion or by active transport via particular ligand/transporter channels and after that may sequester or accumulate in intracellular parts such as the vacuoles^74^.

**Figure 2:**
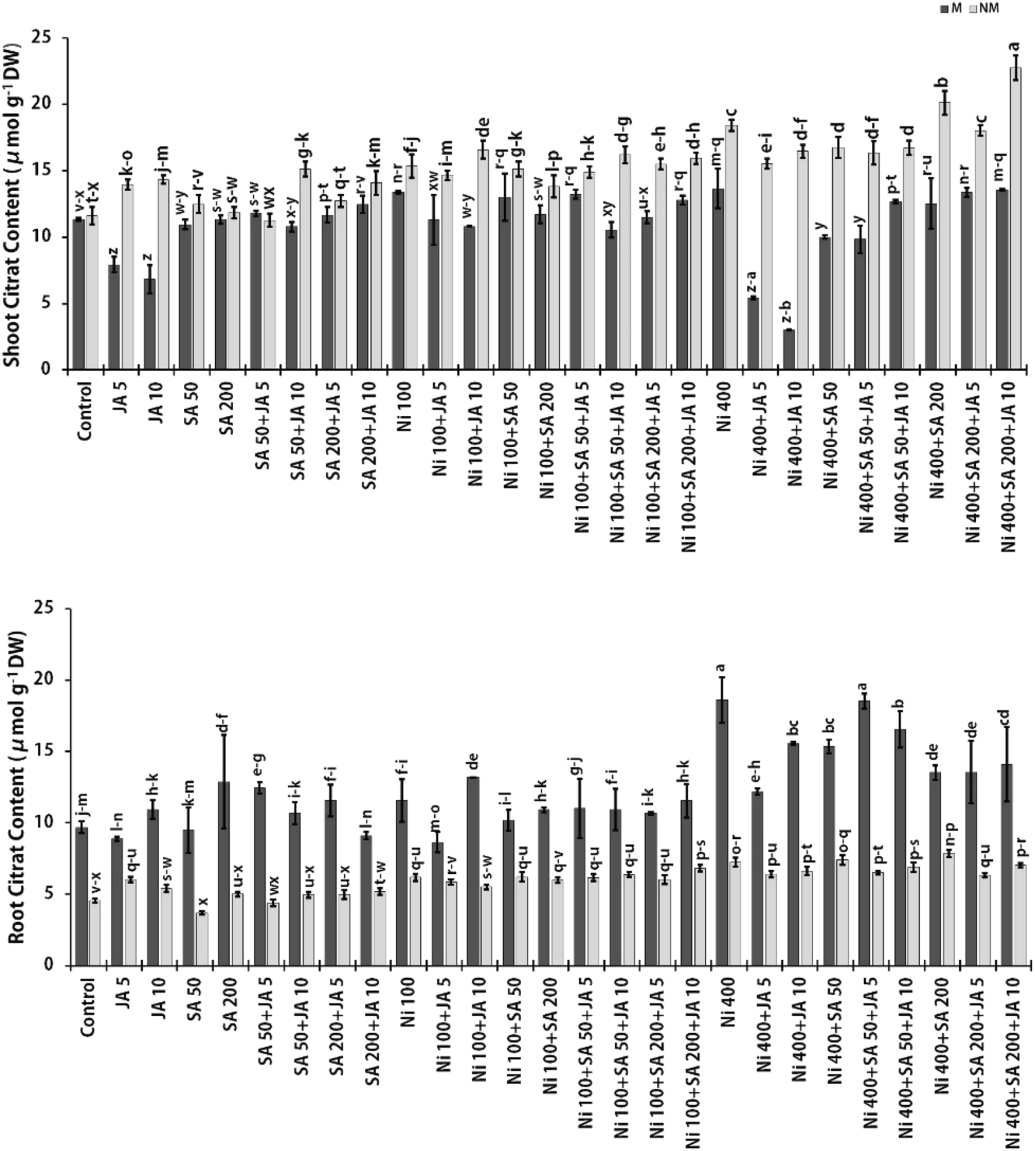
Changes in CA contents in the presence of SA and JA in two parts of populations (NM and M) of *A. inflatum* plants stressed with various doses of Ni. All values in the figure are presented as mean ± SE (*n* = 3).

**Figure 3:**
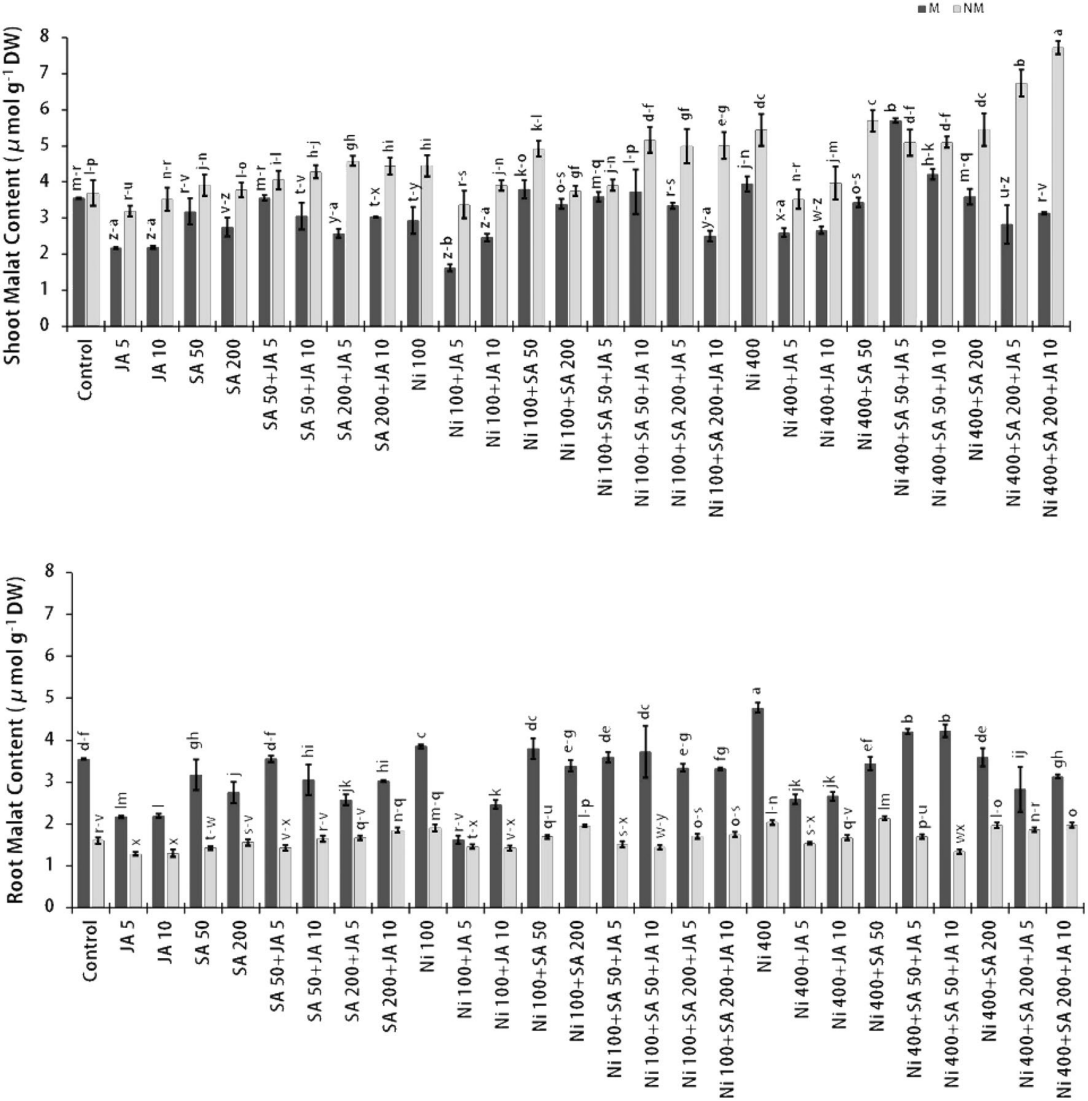
Changes in MA contents in the presence of SA and JA in two parts of populations (NM and M) of *A. inflatum* plants stressed with various doses of Ni. All values in the figure are presented as mean ± SE (*n* = 3).

The content of OA including MA and CA as Ni chelators that role in its transfer and as well as stored in roots and shoots of M and NM populations of *A. inflatum* are displayed in Figs. 2 and 3. Interestingly, the CA and MA amounts in both populations' plant roots and shoots were raised due to an increment in Ni concentration in the plant growth medium. According to the results obtained from this experiment, in plant roots exposed to 400 μM Ni, the CA content was increased by 47.9% and 37.2% (Fig. 2), and as well as the MA content was augmented by 25.5% and 21.2% (Fig. 3), in the M and NM populations, respectively, in comparison to the untreated plant groups. As it is known, CA is synthesized in plants by citrate synthase enzyme. Compared to MA and oxalate, this OA has a greater tendency for metal ions such as Ni^2+^ and Cd^2+^, although, its chief role is to chelate Fe^2+^^73^. Furthermore, in the shoot regions of plants under 400 μM Ni stress, the content of CA and MA augmented by 17.0% and 37.0% in the M population and by 10.0% and 32.0% in the NM population, respectively, relative to the unexposed plants group. Similarly, Amari et al. (2016) demonstrated a significant rise of MA content in xylem sap in Ni-stressed *B. juncea* and *Mesembryanthemum crystallinum*. While CA content augmented in the xylem sap of *B. juncea* alone led to the accumulation of a greater Ni amount than *M. crystallinum*^75^. According to our results, in the presence of a high dose of Ni, greater amounts of Ni were accumulated in the shoots than in the roots of both populations of *A. inflatum*. These outcomes recommend that CA and MA may play a role in long-distance Ni transport and can also perform as intracellular chelators capable of binding Ni in the cytosol or subcellular parts, hence preventing the harmful activity of free HM ions^75^. In contrast to our results, Pietrini et al. (2015) revealed that at higher Ni doses (150 μM), both oxalic and CA could not prevent the deleterious effects on Amaranthus plants at the physiology-biochemical points initiated by this metal. Although in Ni-exposed plants, the content of CA in plant leaves increased relative to the control, with the maximum amount against 25 μM NiCl_2_, in plant roots, the high amount of CA was observed only at 150 μM NiCl_2_. Also, the MA content has not changed in the root and shoot of Amaranthus plants exposed to Ni stress, indicating that this OA in this species may play a minor role as a metal chelator^76^. Dresler et al. (2014) found that in maize seedlings exposed to 50 and 100 μM Cd and Cu stress, CA and MA (at 100 μM Cd) perhaps have a role in Cd transport and sequestration in old leaves, whereas MA is involved in the detoxification of Cu at 50 μM dose. This indicates that CA is one of the Cd chelators involved in the translocation of Cd to older parts of the plant^77^.

A reducing trend in MA and CA contents in the existence of SA or JA at 400 μM Ni was observed in most shoots and roots of the M and NM plants against 400 mM Ni in comparison plants stressed to 400 μM only. Nevertheless, the usage of exogenous SA at the highest dose (200 μM) created an increase only in the content of CA (1.1-fold) in two parts of the NM population plants exposed to 400 μM Ni compared to plants under Ni (400 μM) alone. The OAs contents in two plants' parts of the NM populations under different Ni doses and the simultaneous impact of SA and JA showed significant increases. Thus, in plants treated with 400 μM Ni + 200 μM SA + 10 μM JA, CA content increased by 1.54- and 1.96-fold, and MA content increased by 1.23- and 2.1-fold in roots and shoots, respectively, relative to the untreated plants. In the same treatment, the enhancing effect was observed only on the CA level, with a rise in the shoot (1.2-fold) and root (1.45-fold) of the M population to the untreated plants. In the M population, the maximum increase trend was determined by 1.9 times in root CA content and 1.6 times in shoot MA content in the treatment of 400 µM Ni + 50 µM SA + 5 µM JA compared to the control group. Also, the highest MA amount in M population plants was recorded in the roots under the influence of 400 µM Ni + 50 µM SA + 10 µM JA, with an increase of 1.2 times compared to untreated plants. However, concerning plants treated with 400 μM Ni alone, the maximum MA (29.6%) and CA (19.0%) content were observed at the treatments of 400 μM Ni + 200 μM SA + 10 μM JA in the shoot parts of NM plants. By inducing resistance pathways in M and NM populations under Ni stress, SA and JA led to an increase in the amounts of CA and MA in different parts of these plants to chelate with free Ni ions and sequester them in intracellular organelles such as vacuoles. The mechanism of chelation and separation of Ni by OAs can prevent the induction of oxidative stress and serious damage to the photosynthetic apparatus and plant cells^30,78^. Accordingly, tomato seedlings exposed to Pb stress indicated that Pb at 0.75 mM increased the content of CA (56.6%), MA (40.9%), fumaric acid (29.1%), and succinic acid (25.2%) over the unstressed plants. In these plants, the treatment of 100 nM JA + 0.75 mM Pb elevated CA (19.5%), MA (38.3%), fumaric acid (49.7%), and succinic acid (41.4%) in comparison to the seedlings exposed to Pb (0.75 mM) alone. It was found that the use of JA changed the expression of enzyme genes related to the metabolism of OAs in tomato seedlings under lead stress under toxic doses of lead, accordingly the amount of CA synthase and MA synthase enzymes decreased. Still, seedlings exposed to Pb (0.75 mM) with JA (100 nM) detected an up-regulation of the expression of fumarate hydratase and succinate dehydrogenase enzymes^79^. In line with our findings, Kohli (2018) reported increased CA, MA, succinic acid, and fumaric acid contents in *B. juncea* under stress to Pb (0.75 mM). In this plant, the presence of SA (1 mM) and 24-epibrassinolide acid (EBL) (10^–7^ M) alone led to a greater rise in the amounts of these OAs, as well as the simultaneous treatment with EBL + SA showed the greater effect than these treatments alone in increasing the amounts of these compound in Pb-treated seedlings. For example, the authors demonstrated 123% and 141% increases in CA and MA contents, respectively, under treatment with Pb + EBL + SA in stressed plants^80^. However, OAs such as CA and MA, similar to amino acids, have a role in Ni uptake through the formation of Ni-OA complexes by the roots and also cooperate with processes of root-to-shoot translocation and Ni precipitate in cell organelles such as vacuoles^40,72^.

### Histidine (His) concentration

**Figure 4:**
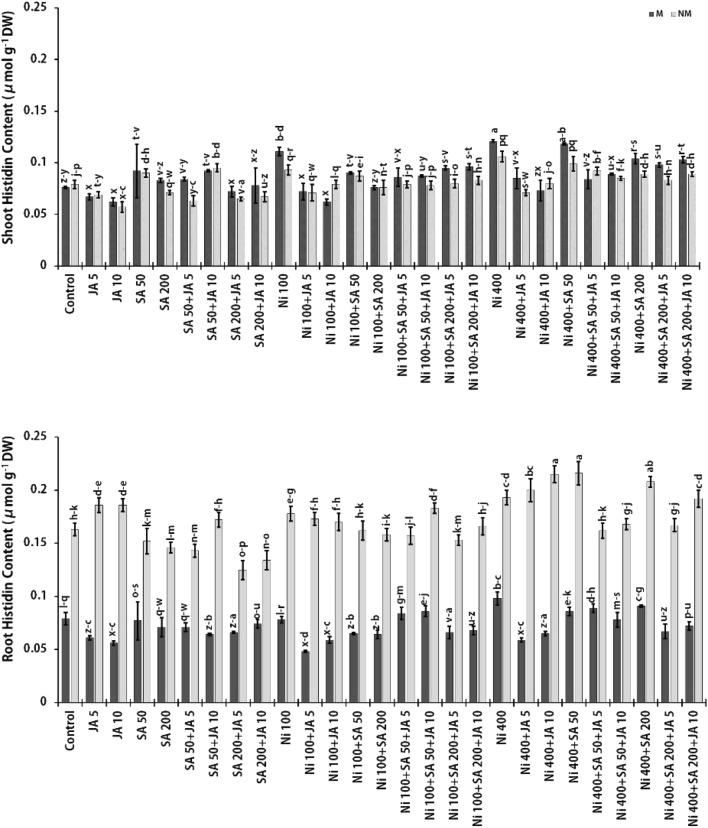
Changes in His contents in the presence of SA and JA in two parts of populations (NM and M) of *A. inflatum* plants stressed with various doses of Ni. All values in the figure are presented as mean ± SE (*n* = 3).

Different amino acids such as His, alanine, asparagine, proline, arginine, methionine, glycine, serine, cysteine, lysine, and glutamic acid, are known to accumulate at high doses against HMs (Ni, Pb, Cr, Zn, Cd) stress to preserve plants from the toxic impacts of certain metals^81^. To protect and tolerate the stressed plants, amino acids can act as compatible osmolytes, pH regulators and ROS detoxifiers as well as acting as nitrogen or carbon resources for the synthesis of certain enzymes and precursors of various secondary metabolites for instance flavonoids and lignin^55^. For example, His has been involved in the detoxifying of HMs in Ni-tolerant plants by its chelation^82^. Therefore, in the current study, the His levels related to the roots and shoots of both populations of *A. inflatum* were analyzed, as shown in Fig. 4. As be shown, high Ni doses enhanced the content of His in stressed plants. Its content was enhanced by 1.24- and 1.18-fold in the roots and incriminated by 1.59- and 1.34-fold in the shoots of the M and NM plants, respectively, with respect to the unstressed plants. It has been established that His has a high tendency to link with metals. Likewise, the free amino acids and proteins with (metal's coordination residues) have a partly high correlation constant (Log K: 8.7) for Ni^83^. According to Dalir, and Khoshgoftarmanesh’s hypothesis, Ni absorption in the presence of His may occur through two pathways: (I) Ni absorption action by the root may be individually from His absorption, however, the existence of free His around roots indirectly induces Ni absorption by chelating with Ni inside the apoplastic or symplastic spaces of the root. (II) Ni(His) ligands are directly absorbed by the root, and thus, His may enhance Ni absorption through specific His- or Ni(His)-transporters^30^. Similarly, Ali et al. (2009) indicated the increasing concentration of His, serine, and cysteine in the xylem sap of *B. napus* cultivars exposed to various doses of Ni. In that research, the higher amount of His was attributed to the decontamination of Ni with increased binding to His, serine, and cysteine, and thus indicated Ni tolerance in *B. napus*^84^. Another study demonstrated an increase in the amount of His in the roots and leaf rosettes of *Matricaria chamomilla* against Ni stress, with a fourfold increase in plant roots at 120 μM Ni^85^. In addition, it was found that more His accumulated in Ni hyperaccumulator *Alyssum lesbiacum* than in non-accumulator *Alyssum montanum* under Ni stress condition^20^. Furthermore, Callahan et al. (2007) reported a rise in the amount of His and nicotianamine in response to higher Ni concentrations in *Thlaspi caerulescens* as a serpentine population^86^.

According to Fig. 3, the content of His in the shoots of two populations of *A. inflatum* under 400 μM Ni stress, in the presence of high doses of SA [in M population (14.0%), NM population (16.0%)] and JA [in M population (7.30%), NM population (24.5%)], exhibited a significant reduction over the plants stressed with Ni at a high dose. However, contradictory results were obtained in the root part of these plants. Besides, the simultaneous effect of SA and JA in combination at different concentrations in Ni-stressed plants caused a decrease in His content relative to the plants stressed with Ni alone. Although, compared to the unstressed plants, treatments of 400 μM Ni + 50 μM SA + 5 μM JA in M species roots by 1.13-fold and the treated group of 400 μM Ni + 200 μM SA + 10 μM JA in shoots of the M (1.36-fold) and NM (1.13-fold) populations and as well as the root of NM population by 1.2-fold caused an increase in the content of His. Considering that in the presence of SA and JA, the reduction of Ni accumulation in the shoots of the two populations under Ni stress occurred simultaneously with the reduction of His content. These results show the probable role of SA and JA in modulating Ni stress by reducing the expression of genes related to the biosynthesis of Ni chelators (such as His and OAs) and transporters involved in its uptake and transfer from root to shoot^83^. Similarly, the results obtained by Ahmad et al. (2021) indicated increasing proline and glycine betaine (GB) accumulation in chickpea plants under Cd stress supplemented with JA and GA_3_. They reported that the combination of JA and GA_3_ in stressed plants (Cd + JA + GA_3_) remarkably augmented proline and GB content by 6.08 times and 7.64 times increase, respectively, compared to control plants^70^. Furthermore, similar to our results, Bali et al. (2019) revealed that the treatment of tomato seedlings with 100 nM JA + 0.75 mM Pb reduced levels of His, cysteine, aspartic acid, isoleucine, leucine, and GABA (non-protein amino acid), while increased the content of other amino acids compared to plant stressed with 0.75 mM Pb alone. They pointed to the amino acids' role in nitrogen (N) metabolism that regulates the protein metabolism, re-mobilization of N, discount of nitrate and uptake of ammonium and nitrate^79^. Likewise, Zanganeh et al. (2019) reported that the usage of SA on the seeds of the *Z. mays* plant, as a pre-treatment, led to a reduction in the amount of His in plant shoots and roots under Pb treatment, while the application of sodium hydrosulfide (NaHS) on the plant seeds caused a decline in the content of roots His and an augmentation in the level of His in shoots. However, according to the authors' results, SA accumulated other amino acids similar to alanine, tyrosine, valine, tryptophan, leucine, and isoleucine. They explained that the decrease of amino acid levels by pre-treatment with SA and NaHS could be considered as a contribution to the beginning of adaptive processes with the unfavorable impact of Pb. Amino acids play precursor roles in synthesizing stress-related proteins and defensive metabolites against plant stress. On the other hand in their study, the increased content of His was attributed to the production of de novo and N storage to inhibit ammonium toxicity and as a result the increase in the contents of His and arginine amino acids, which are appropriate for N storage owing to the existence of binary amides in their structure^78^. These results were in line with our results, which observed that SA declined His levels in the shoots of both populations and root of the M population under Ni stress. However, the using exogenous phytohormones such as JA and SA can cause an increment in the content of endogenous amino acids in plants, resulting in improved plant tolerance to abiotic stress like HMs. This is achieved by enhancing plant metabolism through the regulation of membrane permeability, the activation of enzymatic process, the production of osmolytes, and the uptake of some ions^87^.

### Principal component analysis (PCA)

**Figure 5:**
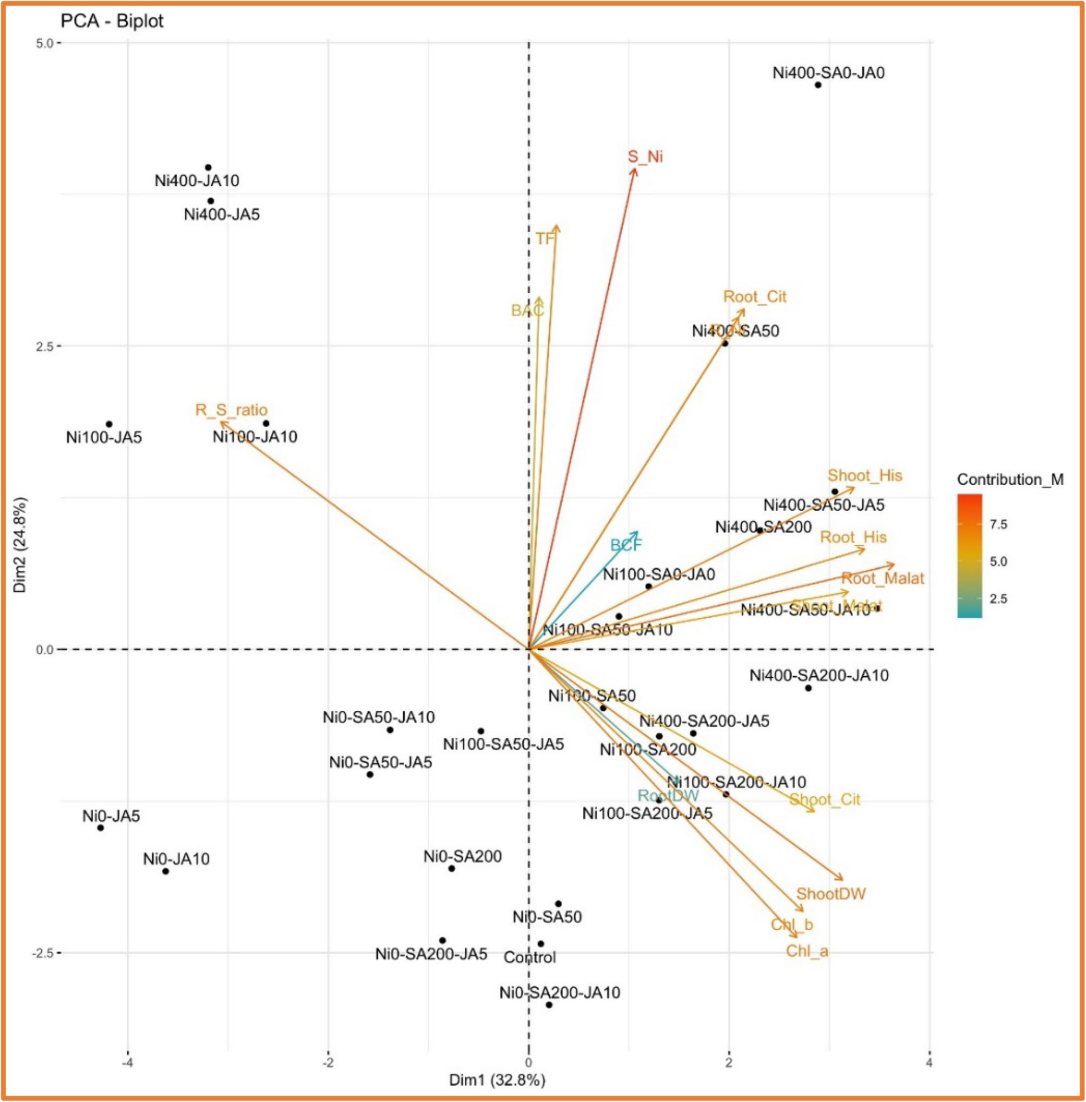
Biplot diagram illustrating the principal component analysis (PCA) for the variables studied in the M population. Treatments are depicted in black, while the studied variables are represented by arrows. ShootDW: shoot dry weight; RootDW: root dry weight; R_S ratio: ratio of root/shoot dry weight; Chl a: chlorophyll *a*; Chl_b: chlorophyll *b*; S_Ni: shoot Ni content; R_Ni: root Ni content; Shoot_His: shoot histidine content; Root_His: root histidine content; Shoot_Cit: shoot citric acid content; Root_Cit: root citric acid content; Shoot_Malat: shoot malic acid content; Root_Malat: root malic acid content; BCF: biological concentration factor; BAC: Bioaccumulation coefficient; TF: translocation factor.

**Figure 6:**
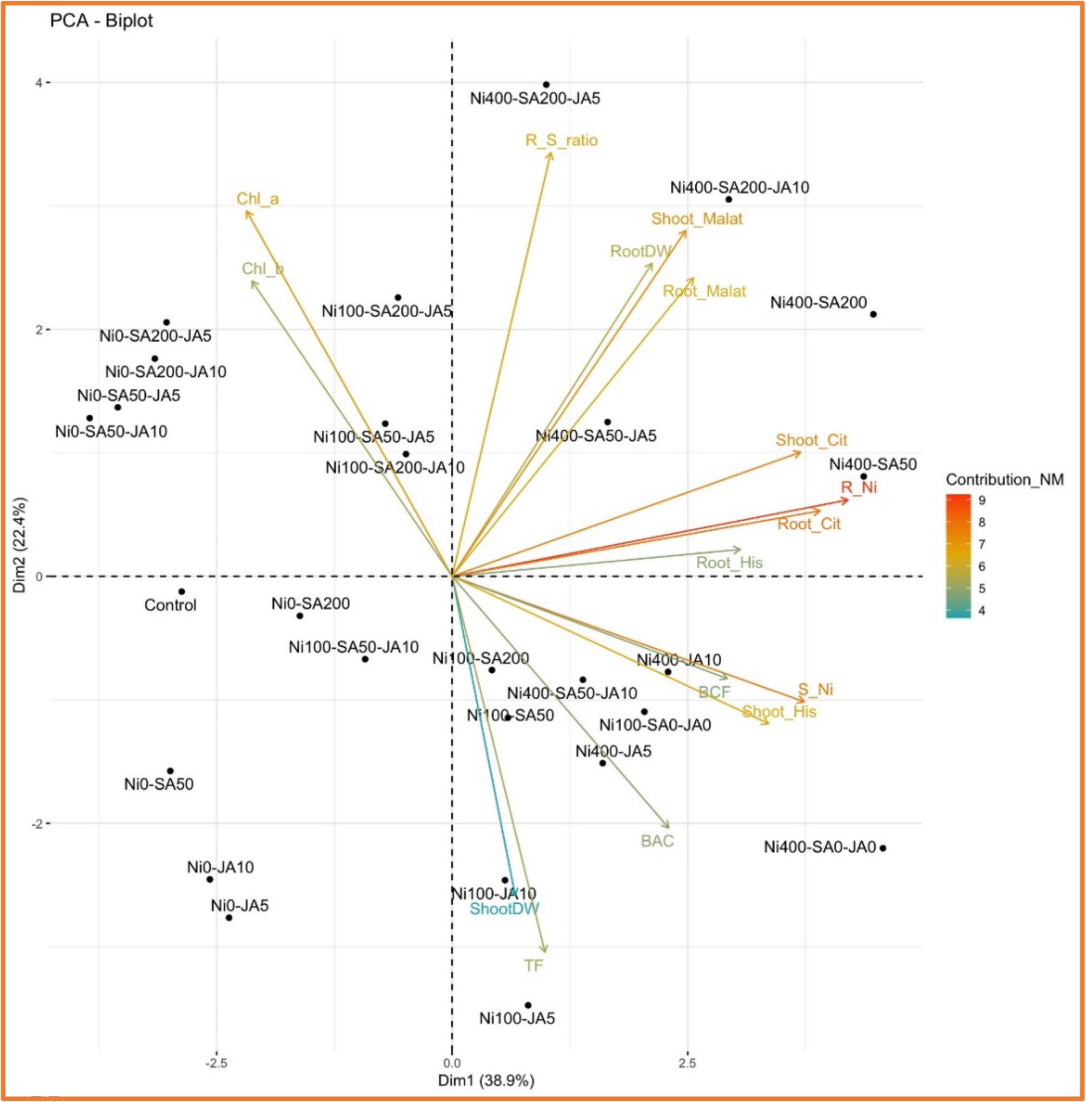
Biplot diagram illustrating the principal component analysis (PCA) for the variables studied in the NM population. Treatments are depicted in black, while the studied variables are represented by arrows. ShootDW: shoot dry weight; RootDW: root dry weight; R_S ratio: ratio of root/shoot dry weight ;Chl a: chlorophyll *a*; Chl_b: chlorophyll *b*; S_Ni: shoot Ni content; R_Ni: root Ni content; Shoot_His: shoot histidine content; Root_His: root histidine content; Shoot_Cit: shoot citric acid content; Root_Cit: root citric acid content; Shoot_Malat: shoot malic acid content; Root_Malat: root malic acid content; BCF: biological concentration factor; BAC: Bioaccumulation coefficient; TF: translocation factor.

**Figure 7:**
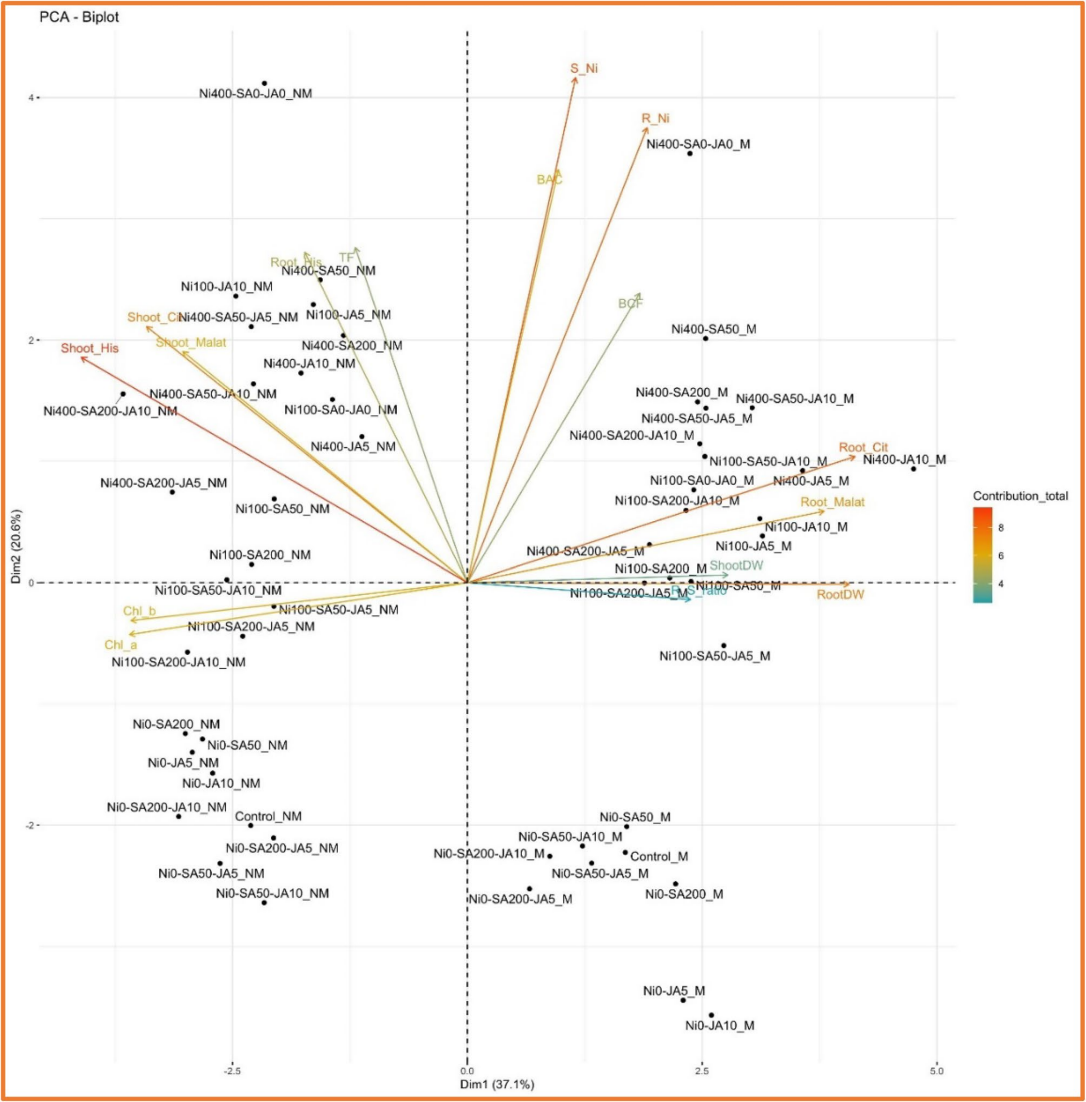
The biplot diagram displays the outcomes of Principal Component Analysis (PCA) for the studied variables across both populations of M and NM. Treatments are depicted in black, while the studied variables are represented by arrows. ShootDW: shoot dry weight; RootDW: root dry weight; R_S ratio: ratio of root/shoot dry weight ;Chl a: chlorophyll *a*; Chl_b: chlorophyll *b*; S_Ni: shoot Ni content; R_Ni: root Ni content; Shoot_His: shoot histidine content; Root_His: root histidine content; Shoot_Cit: shoot citric acid content; Root_Cit: root citric acid content; Shoot_Malat: shoot malic acid content; Root_Malat: root malic acid content; BCF: biological concentration factor; BAC: Bioaccumulation coefficient; TF: translocation factor.

Using PCA we investigate the interrelationships within a set of variables and elucidated them via diverse biplots. In our investigation of the correlation between variables and traits across two distinct populations of *A. inflatum*, we employed PCA independently for each population and collectively across both populations (Figs. 5, 6, 7). The first two principal components of PCA accounted for (57.7%), (57.6%) and (61.3%) of the variance when analyzing both populations collectively or when examining populations M and MN separately. Furthermore, PCA plots demonstrated a similar trend in both M and NM plants in response to the presence of SA and JA in plants under Ni stress. The PCA results revealed that in the M (Fig. 5) and NM (Fig. 6) populations, in the presence of SA and JA supplements under Ni toxicity, there was an approximate positive correlation between plant biomass, amount of Ni and the levels of His, MA, SA, and phytoremediation indices (TF, BAC, BCF). This pattern was also seen in the common PCA plot between the two populations (Fig. 7). Interestingly, the highest correlation between phytoremediation parameters and Ni levels of roots and shoots was observed in population M. The pattern was also observed in the NM population. On the other hand, the lowest correlation value between chlorophyll (*a*, *b*) levels and phytoremediation parameters was detectable in all PCA plots. In total, the findings of this study and also the PCA plots showed that both populations (M, NM) follow a similar pattern in the presence of SA and JA alone or together (SA + JA) in reaction to high Ni doses. It can also be stated that although SA and JA, especially the presence of both of them together led to the activation of plant resistance mechanisms against Ni toxicity and decreased phytoremediation values (TF, BAC, BCF) in both populations (M, NM) exposed to high doses of Ni, however, the values of phytoremediation parameters were still higher than one. Finally, it is possible to confirm the effective role of both populations of *A. inflatum* as useful plants (Ni-hyperaccumulators) in remediation of Ni from contaminated sites. Although there have been extensive debates about the role of SA and JA in HM stress conditions, according to our knowledge, no study has been documented the impacts of these phytohormones simultaneously on phytoremediation parameters. Therefore, this study is an opening to clarify and evaluate the effective role of SA, JA, or/SA + JA in phytoremediation mechanisms in two populations of *A. inflatum*.

## Conclusion

Phytoremediation is an effective internal mechanism for the absorption of HMs and their sequestration in various plant tissues. This process enables the removal of HMs from contaminated sites at a faster rate and succeeding harvesting at a lower cost. On the other hand, phytohormones such as SA and JA improve the physio-biochemical processes, increasing plant tolerance to adverse environmental conditions and protecting against various stressors, including HMs. Our findings indicate that SA and JA moderated the deleterious impacts of Ni on physiological parameters. They decreased the levels of CA, MA and His in both populations, thereby modulating plant tolerance to Ni stress. Although the values of phytoremediation indices (TF, BAC, BCF) were greater than one in both populations exposed to Ni (400 μM), a decreasing trend in these values was observed in the presence of SA and JA. Overall, the results revealed that these phytohormones can reduce the phytoremediation potential in the two populations through various mechanisms, although these values were still greater than one. These results confirm the role of both *A. inflatum* populations as an effective plant for phytoremediation in Ni-contaminated sites. Overall, a deeper understanding of the effective remediation mechanisms and the critical role of phytohormones in plant resistance strategies can provide a clearer perspective on plant response to varieties of unfavorable environmental conditions and the proper use of plants.

## Data Availability

The authors declare that the data supporting the findings of this study are available within the paper. Should any raw data files be needed in another format, they are available from the corresponding author upon reasonable request.

## Abbreviations

BAC: Bioaccumulation coefficient
BCF: Biological concentration factor
CA: Citric acid
DW: Dry weight
HM: Heavy metal
His: Histidine
JA: Jasmonic acid
MA: Malic acid
Ni: Nickel
OA: Organic acid
PGR: Plant growth regulator
SA: Salicylic acid
TF: Translocation factor
